# Ascorbic Acid-Caused Quenching Effect of Protein Clusteroluminescence Probe: The Fast Fluorescent Detection of Ascorbic Acid in Vegetables

**DOI:** 10.3390/molecules28052162

**Published:** 2023-02-25

**Authors:** Jiying Song, Xinyan Guo, Haiying Chen, Yunge Tang, Lei Han

**Affiliations:** College of Chemistry and Pharmaceutical Sciences, Qingdao Agricultural University, 700 Changcheng Road, Qingdao 266109, China

**Keywords:** biomacromolecule, clusteroluminescence, clusterization-triggered emission, fluorescent detection, bovine serum albumin, ascorbic acid, fluorescence quench

## Abstract

It is interesting and meaningful to explore fluorescent probes for novel rapid detection methods. In this study, we discovered a natural fluorescence probe, bovine serum albumin (BSA), for the assay of ascorbic acid (AA). Due to clusterization-triggered emission (CTE), BSA has the character of clusteroluminescence. AA shows an obvious fluorescence quenching effect on BSA, and the quenching effect increases with increasing concentrations of AA. After optimization, a method for the rapid detection of AA is established by the AA-caused fluorescence quenching effect. The fluorescence quenching effect reaches saturation after 5 min of incubation time and the fluorescence is stable within more than one hour, suggesting a rapid and stable fluorescence response. Moreover, the proposed assay method shows good selectivity and a wide linear range. To further study the mechanisms of AA-caused fluorescence quenching effect, some thermodynamic parameters are calculated. The main intermolecular force between BSA and AA is electrostatic, presumably leading to the inhibiting CTE process of BSA. This method also shows acceptable reliability for the real vegetable sample assay. In summary, this work will not only provide an assay strategy for AA, but also open an avenue for the application expansion of CTE effect of natural biomacromolecules.

## 1. Introduction

Ascorbic acid (AA), generally known as vitamin C, is an essential micronutrient that the human body is unable to synthesize for itself and needs to be obtained from the diet [1]. As one of the water-soluble vitamins, it has been extensively identified in fresh fruits, vegetables and human blood. Many pieces of research have demonstrated that AA is of practical significance in the fields of medical treatment, clinical diagnosis and food production [2,3,4,5,6,7]. For example, it can not only effectively prevent and treat scurvy but also enhance the human body’s immune function [4]. Moreover, AA can also be used in beauty skin protection to prevent skin aging by promoting the synthesis of collagen in the body [8]. The content of AA has also acted as a key biological marker for diagnosing health disorders and the quality control of health products [9,10]. Additionally, AA is a type of unstable substance, which can easily combine with oxygen to passivate metal ions. So, the inherent properties of AA can effectively inhibit the browning of fruits and vegetables and change the flavor of food and AA is often used as an antioxidant in the food industry to prevent discoloration or flavor change during processing [11]. Therefore, the content of AA in food and beverages is also a quality indicator of antioxidant capacity [11]. Besides, AA is a nutritional enhancer, and the appropriate uptake can promote dietary intake [1,12]. It is altogether necessary to accurately detect AA in fruits, vegetables and other food products.

At present, several analysis methods are commonly applied for the detection of AA, such as chromatography [13,14], redox titration [15], spectrophotometry [16,17,18] and electrochemistry [19,20,21,22]. These methods for the determination of AA have their own characteristics [23]. Chromatographic methods, such as high performance liquid chromatography, characteristically possess high sensitivity and accuracy for the determination of AA, but expensive equipment and professional operating personnel are required [23,24]. The redox titration method is simple to operate, but the determination of the titration endpoint is difficult to accurately achieve [23,25]. Although the operation of spectrophotometry has a simple detection process, the results are easily disturbed by the color of the sample itself [23,26]. The electrochemical techniques are sensitive, but cannot obtain accurate results in complex samples [23,27]. Hence, these AA assay methods cannot simultaneously satisfy the need for rapid assays, anti-interference capabilities and high sensitivity. Recently, the fluorescence detection method has been extensively applied in the field of analytical chemistry, due to its fast response, visualization, convenience and high sensitivity [28,29,30]. For the fluorescence detection method, the fluorescence probes play a critical role in the effective recognition of small molecular targets [31]. Although some routine fluorescent probes have been widely employed, a complex design and synthesis were required [32,33]. Compared with synthesized fluorescent probes, natural fluorescent probes show enormous advantages of low-cost and easy accessibility [34]. However, natural fluorescent probes have been rarely reported as being able to recognize AA. Therefore, it is of great significance to explore natural fluorescent probes for the fluorescent detection of AA.

At the beginning of this century, it was found that the luminescence behavior in non-conjugated molecules could be attributed to the properties peculiar to the non-conjugated compounds [35,36,37]. Tang’s team defined this type of luminescence as clusteroluminescence and this process as clusterization-triggered emission (CTE) [35,38,39]. Clusteroluminescence is caused by the aggregation of the unconjugated molecules under UV irradiation, while diluted solutions do not emit light [40,41,42]. Using this photoluminescence phenomenon, a number of applications can be developed, including biosensors and the visualization of biological processes [43,44]. This phenomenon has also been found in many natural compounds, such as starch, cellulose and protein [35,45]. Typically, serum albumin, as the most plentiful protein in plasma, plays an indispensable role in the biological system [46]. Bovine serum albumin (BSA) has generally been applied as a commercial globulin in bovine serum. BSA was widely used in various fields including the synthesis of nanomaterials [47,48], encapsulation materials [49] and as the block agent of biosensors [50]. Due to good biocompatibility and non-specific absorption, BSA was selected as a fluorescent marker for analytical detection [51]. Although the clusteroluminescence of BSA had been reported [52], the effect of interaction with other molecules on fluorescence had rarely been researched. BSA composites were mostly employed in the detection of small molecules, but they only performed auxiliary functions, such as stabilizing nanomaterials, attaching fluorescent probes and so on [53]. There were few studies on BSA clusteroluminescence probes for fluorescence detection. Thus, it is of great importance and interest to find natural CTE probes for realizing low-cost and rapid fluorescence detection.

Here, BSA was used as a natural protein clusteroluminescence probe to realize the fast and sensitive detection of AA. Interestingly, AA can impact on the clusteroluminescence properties of BSA. Not only does AA have a quenching effect on the fluorescence of BSA, but also the decrease in fluorescence intensity has a positive linear relationship with the concentration of AA. Accordingly, considering the fluorescence quenching effect of BSA by AA, a quick AA fluorescence assay was established. The proposed AA sensing platform based on quenching fluorescence effect on BSA realized the simple, rapid, sensitive and selective detection of AA. Further, the mechanism of the AA-caused quenching effect of BSA was judged by the Stern–Volmer equation and the Lineweaver–Burk plot, indicating the static quenching effect of AA on BSA. Moreover, the thermodynamic parameters of fluorescence quenching were calculated by the Van't Hoff equation, showing the presence of electrostatic forces between molecules. Finally, the developed method was successfully applied to detect AA in practical samples, and satisfactory results were obtained for the determination of three different vegetables. This work not only provides insights for the interaction of CTE probes with other molecules, but also opens an avenue for a fluorescence sensing platform based on the quenching effect of natural clusteroluminescence biomacromolecules.

## 2. Results and Discussions

### 2.1. Effect of AA on Clusteroluminescence of BSA

**Figure 1:**
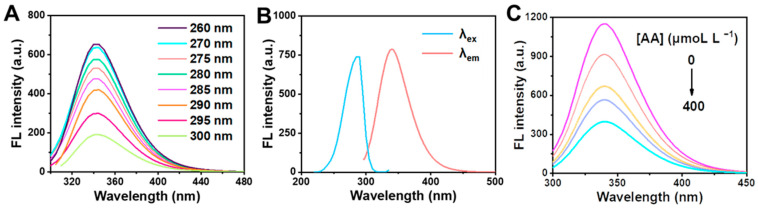
(**A**) Fluorescence emission spectra of BSA at different excitation wavelengths. (**B**) Fluorescence excitation (λ_em_ = 340 nm) and emission (λ_ex_ = 288 nm) spectra of BSA. (**C**) Fluorescence emission spectra of system containing various concentration of AA (0, 40, 100, 200 and 400 μmol L^−1^).

To investigate the influence of AA on clusteroluminescence of BSA, the fluorescence intensity of BSA was analyzed by fluorescence spectra [34]. Firstly, BSA (0.1 mol L^−1^) standard solution was taken into the spectrometer, and different excitation wavelengths were set for emission spectrum scanning to determine the fluorescence emission wavelength of BSA. Figure 1A shows that the maximum emission wavelength of BSA was about 340 nm at different excitation wavelengths. Subsequently, the maximum excitation wavelength was 288 nm at the maximum emission wavelength by scanning the excitation spectrum. Therefore, the maximum emission wavelength of BSA was identified at 340 and the maximum excitation wavelength was determined at 288 nm. Figure 1B showed that the maximum absorption wavelength was obtained at 278 nm from the fluorescence excitation and emission spectra of BSA according to ultraviolet spectrum scanning. In addition, the various concentrations of AA (0, 40, 100, 200 and 400 μM L^−1^) were added to the BSA, and the fluorescence spectrum scanning was performed to analyze the effect of AA on BSA clusteroluminescence. It can be seen from Figure 1C that the position of the fluorescence emission peak of BSA is generally unchanged after the addition of AA, and the fluorescence intensity of BSA gradually declines with the increase in AA concentration. The results suggest that AA has a quenching effect on BSA.

### 2.2. Condition Optimization and Selectivity Assay

**Figure 2:**
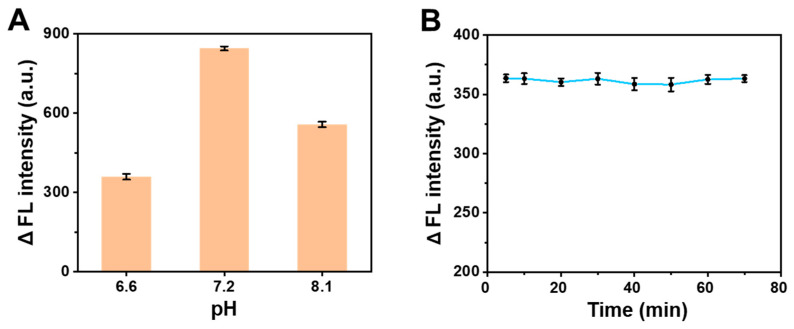
(**A**) Optimization of pH on the change in fluorescence intensity of the AA-BSA system. (**B**) Optimization of reaction time on the change in fluorescence intensity of the AA-BSA system.

To optimize the reaction condition for AA detection, the fluorescence intensity of the AA-BSA system was measured at different pH and reaction times. Based on the factory specification of the BSA, the pH-dependent condition was used in the pH range of 6.5–7.2. A too high or too low level of pH had an influence on the structure and fluorescence of the BSA, affecting the reliability of the study, thus AA was detected at pH 6.6 and 7.2. To further illustrate the optimal level at pH 7.2, the detection of AA was also performed at pH 8.1. Owing to the preparation of the PBS (pH = 6–8.5) comprising a mixture of NaH_2_PO_4_ and Na_2_HPO_4_ the actual pH could not be accurately controlled, so the used pH value was the actual measured pH value. The pH levels of 6.6, 7.2 and 8.1 were selected based on the above reasons. As shown in Figure 2A, we found that the pH had different influences on the decreasing value of fluorescence intensity (ΔF) after the same reaction time in the AA-BSA system, and ΔF was the largest at pH 7.2. The above-selected pH -already proves that CTE was affected by pH, and the optimal pH (7.2) was applied for the subsequent experiments.

The interaction between organic small molecules and macromolecules required a certain time, which can affect their binding sites and binding constants. So, the trend plot of fluorescence intensity at different reaction times was obtained (Figure 2B). The fluorescence intensity of the solution changed slightly and the ΔF remained nearly stable within the range of 5–70 min. To save time, 5 min was selected as the time to determine the interaction between BSA and AA.

**Figure 3:**
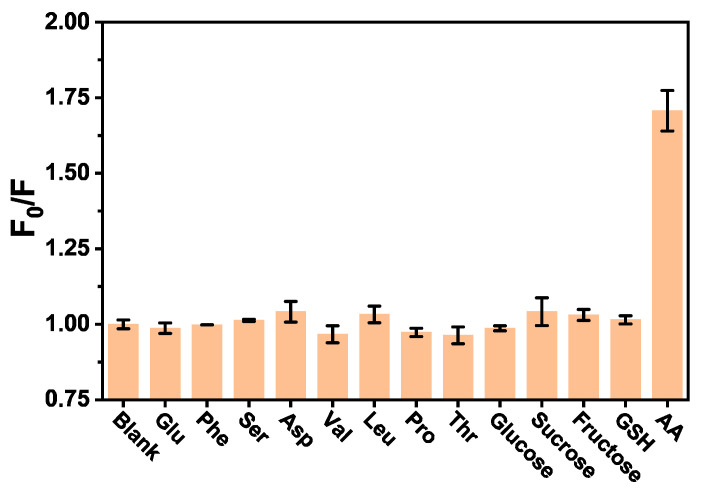
Selectivity of the fluorescence sensor for AA detection.

To research the selectivity of the AA assay method, some interferents including amino acids (glutamic acid (Glu), phenylalanine (Phe), serine (Ser), aspartic acid (Asp), valine (Val), leucine (Leu), proline (Pro), threonine (Thr)), saccharide (glucose, sucrose, fructose) and glutathione (GSH) were used to replace AA in the same concentration under the same conditions. As shown in Figure 3, only AA could provide significant fluorescence responses signal of the system, while other substances in the experiment had no obviously influence on the fluorescence change of BSA. The results manifest that the BSA fluorescent probe has high selectivity for AA assay. Thus, this proposed method based on the fluorescence quenching of BSA by AA would be potentially feasible for the detection of AA in real samples.

### 2.3. Mechanism Study of Fluorescence Quenching of BSA by AA

**Figure 4:**
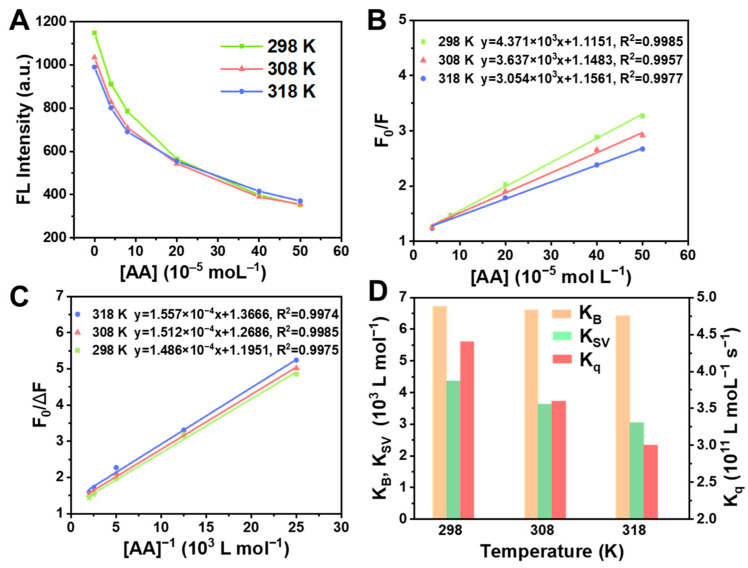
(**A**) Effect of temperatures on fluorescence intensity. (**B**) Stern–Volmer plots of fluorescence quenching of BSA by AA at three different temperatures. (**C**) Lineweaver–Burk curves of fluorescence quenching of BSA by AA at three different temperatures. (**D**) K_B_, K_SV_ and K_q_ values at three different temperatures.

Under the optimized pH and reaction time, the influence of the different temperatures on the fluorescence intensity of BSA at different AA concentrations was studied by fluorescence spectra. As illustrated in Figure 4A, the fluorescence intensity of the solution gradually declined with the increase in temperature at the same AA concentration. The results showed that the temperature had an effect on this reaction system, and the fluorescence quenching degree of BSA by AA was enhanced with the increase in temperature.

To research the mechanism of the fluorescence quenching of BSA by AA, the quenching process was further discussed by analyzing the data obtained. Fluorescence quenching, also known as extraction quenching, is a phenomenon whereby the fluorescence intensity and lifetime of fluorescent molecules are decreased for some reason, and the substance that causes the quenching of fluorescent molecules is called quenchers. Dynamic quenching refers to the process of diffusion-related interactions between the quenchers and the excited-state fluorescent molecules. Static quenching is a process in which the quencher and the fluorescent molecules form a non-luminescent complex in the ground state. The processes of the fluorescence quenching, including dynamic quenching and static quenching, which conformed to the Stern–Volmer equation:

F_0_/F = 1 + K_SV_ c (1)

where F_0_ and F are the fluorescence intensity before and after adding quencher; c expresses the quencher concentration (mol L^−1^); K_SV_ designates Stern–Volmer quenching constants (L moL^−1^). According to Equation (1), F_0_/F has a linear relationship with the concentration of the AA. The Stern–Volmer plot at 298, 308 and 318 K is shown in Figure 4B, where F_0_/F is used as the ordinate and the AA concentration is taken as the abscissa, and the slope of the line is K_SV_. The curve is linearly fitted, and the slope of the resulting line is quenching constants. Therefore, the values of quenching constants (K_SV_) at 298, 308 and 318 K were described to be 4.371 × 10^3^, 3.637 × 10^3^ and 3.054 × 10^3^ L moL^−1^, respectively (Figure 4D). With the increase in temperature, K_SV_ gradually decreased, indicating the static quenching.

To further determine the type of quenching between AA and BSA, the bimolecular quenching rate constant (K_q_) was calculated according to the equation:

K_SV_ = K_q_τ_0_ (2)

where τ_0_ is the average lifetime of the fluorescent molecule without the quencher (about 10^−8^ s). As illustrated in Figure 4D, the K_q_ values were 4.371 × 10^11^, 3.637 × 10^11^ and 3.054 × 10^11^ L moL^−1^ s^−1^ at 298, 308 and 318 K, respectively. According to the judgment method of the fluorescence static quenching and dynamic quenching, the maximum diffusion collision constant (2.0 × 10^10^ L mol^−1^ s^−1^) of the quenching agent on biological macromolecules was the baseline, where a K_q_ value much larger than 2 represents static quenching [54,55]. The K_q_ value obtained by the experiment was much larger than 2.0 × 10^10^ L mol^−1^ s^−1^, verifying that the quenching mode between BSA and AA was static, which was consistent with the fluorescence quenching method judged by K_SV_ (Figure 4B).

To further research the interaction of AA with BSA, the Lineweaver–Burk plot was analyzed. In static quenching process, the relationship between fluorescence intensity, quencher concentration, and binding constant (K_B_) can be expressed as the following equation [54]:

1/(F_0_ − F) = F_0_^−1^ + K_D_F_0_^−1^c^−1^ (3)

Equation (3) can be deformed to the following equation:

F_0_/(F_0_ − F) = 1 + K_D_c^−1^ (4)

where K_D_ is the dissociation constant. From Figure 4C, the Lineweaver–Burk plot of AA to BSA fluorescence quenching at 298, 308 and 318 K was obtained. Since the slope of the Lineweaver–Burk equation is K_D_, those at 298, 308 and 318 K are 1.486 × 10^−4^, 1.512 × 10^−4^ and 1.557 × 10^−4^ L moL^−1^, respectively. Subsequently, the binding constants (K_B_) at three different temperatures were calculated to be 6.729 × 10^3^, 6.614 × 10^3^ and 6.423 × 10^3^ L moL^−1^ (Figure 4D), according to the following equation:

K_B_ = 1/K_D_ (5)

It can be seen that the value of the binding constant is larger, indicating that AA has a strong interaction with BSA.

**Figure 5:**
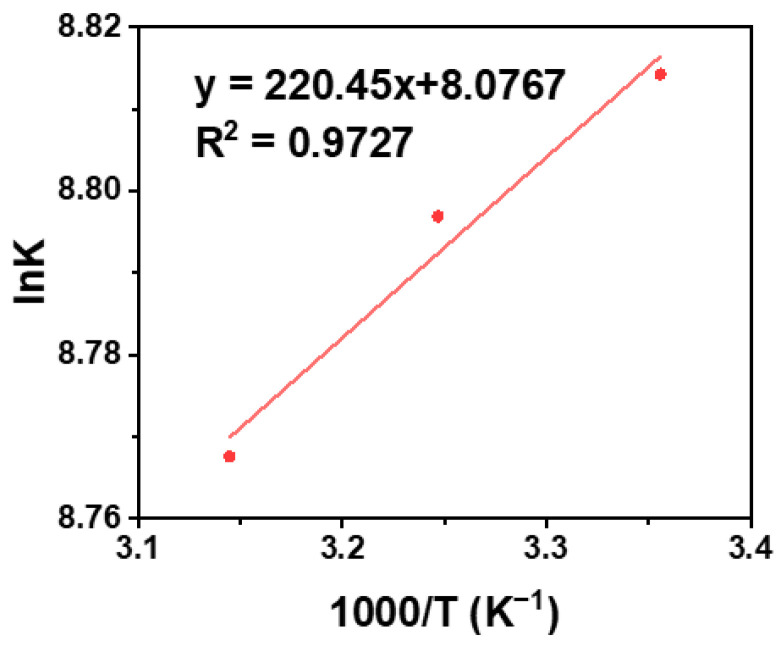
Fluorescence quenching curve of AA-BSA system.

To further explore the type of acting force between BSA and AA, thermodynamic parameters were calculated (Figure 5), because of the interrelationship between the thermodynamic parameters and the acting forces. In general, the binding forces between small organic molecules and biological macromolecules include hydrogen bonds, van der Waals contacts, electrostatic interaction and hydrophobic forces [56]. The forces between BSA and AA were investigated, using the Van’t Hoff equation:

lnK = −ΔH/RT + ΔS/R(6)

where K was calculated, and the gas constant was taken as 8.314 J mol^−1^ K^−1^. The main types of intermolecular forces were judged by the thermodynamic parameters ΔH and ΔS from the Van’t Hoff equation [57,58,59]. The lnK is plotted against 1/T at three different temperatures by the Van’t Hoff equation, where the slope of the curve is ΔH/R, and the intercept is ΔS/R. In Figure 5, the equation of lnK = 220.45 T^−1^ + 8.0767 was obtained by plotting the reciprocal of lnK to temperature, the negative value of enthalpy change (ΔH) is 1.832 KJ moL^−1^ and the positive values of entropy change (ΔS) is 67.15 J moL^−1^ K^−1^ according to this equation. The negative value of ΔH indicated that the process was exothermically reactive, and the positive value of the ΔS showed that the chaos of the reaction system was in the direction of the increase. The results indicate that the acting force between AA and BSA was mainly electrostatic force [58].

**Figure 6:**
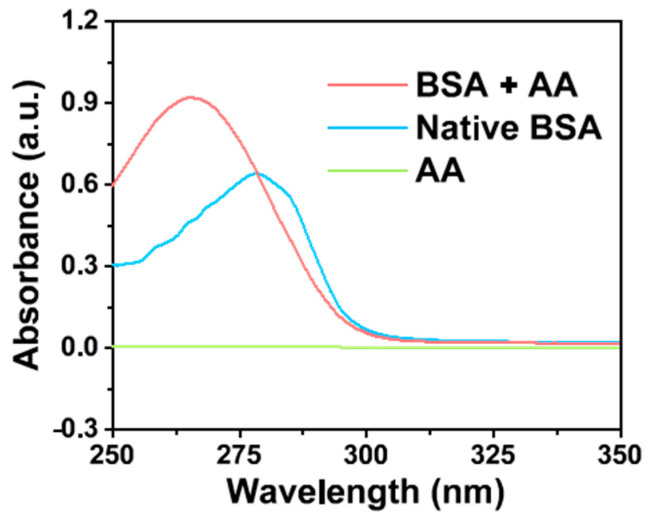
Ultraviolet-visible absorption spectra of BSA in the presence and absence of AA.

The tryptophan (Typ) and tyrosine (Tyr) residues in BSA have adsorption peaks at 278 nm. With the conformation of amino acid residue changes, the corresponding microenvironment and absorption spectra of amino acid are also changed, because the microenvironment of amino acid residues in BSA is determined by the conformation of protein molecules [55]. Therefore, the changes in the absorption peaks reflect the conformational changes of BSA after the binding of small molecules [60]. In this work, to further explore the reaction between AA and BSA, the structural changes of BSA by AA were investigated by ultraviolet-visible absorption spectroscopy. As illustrated in the absorption spectra (Figure 6), the characteristic absorption peak of BSA at 278 nm was caused by the heteroaromatic ring π→π* transition of Trp and Tyr on its peptide chain [61]. Interestingly, after the addition of AA, the absorption peak of BSA increased and its maximum absorption wavelength shifted from 278 to 265 nm. This indicated that the spatial structure of BSA changes during binding, exposing the hydrophobic groups of Trp and Tyr residues surrounding the protein molecule, resulting in an enhancement of the hydrophobic interaction between hydrophobic groups, and the blue-shift of the absorption peak [59]. The results show that there was an interaction between the AA and BSA, and BSA might combine with AA to form a complex. It is deduced that the conformation and microenvironment of BSA changed, which affected the clusteroluminescence of BSA [55].

**Figure 7:**
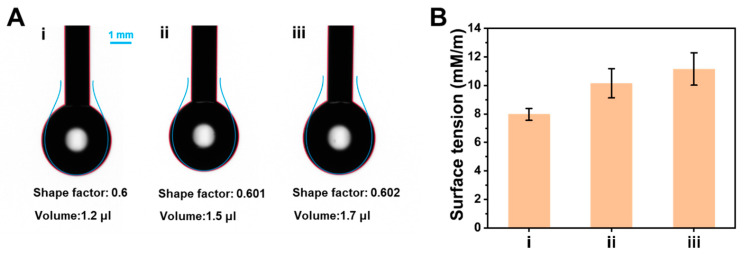
(**A**) Dropping photos and (**B**) corresponding surface tension of different solutions: (i) BSA, (ii) BSA + low concentration of AA (200 μmol L^−1^), (iii) BSA + high concentration of AA (400 μmol L^−1^).

To further study the effect of AA on the conformation of BSA, the surface tension of BSA in the presence or absence of AA was observed by the pendant drop method. As shown in Figure 7A, the volume of the BSA solution drip gradually increased with the increase in AA concentration. Figure 7B indicated that the surface tension of BSA solution with AA was higher than native BSA solution, and the surface tension of BSA gradually increased with the increase in AA concentration. These results suggested that AA decreases the hydrophobicity of BSA [47].

Taken together, BSA, a kind of non-conjugated protein, shows a spontaneous CTE process, and fluoresces under UV irradiation. When AA was added to BSA, AA combined BSA by electrostatic force to form an AA-BSA complex. Therefore, the spatial structure and microenvironment of BSA changed, which caused the static quenching of the clusteroluminescence of BSA. On account of the above results (Figure 4, Figure 5, Figure 6 and Figure 7), the quenching mechanism is summarized as follows.

(1) Owing to electrostatic interactions (Figure 4 and Figure 5), AA was embedded in BSA to form an AA-BSA complex, which changes the microenvironment and conformation of BSA. The polarity of the microenvironment around Tyr and Trp residues changed (Figure 6), resulting in a decrease in hydrophobicity (Figure 7) [59,60]. Moreover, AA was closely combined with Trp, which further reduced hydrophobicity [62];

(2) In the meantime, the BSA protein skeleton became loose (Figure 6) due to the embedding of AA into BSA [63];

(3) Because of the decrease in hydrophobicity and the looseness of the protein skeleton, the aggregation degree of BSA non-conjugated molecular clusters was weakened. This caused intense intramolecular motion and weakened the CTE effect, leading to the clusteroluminescence quenching of BSA [64].

### 2.4. Fluorescent AA Assay Based on BSA

**Figure 8:**
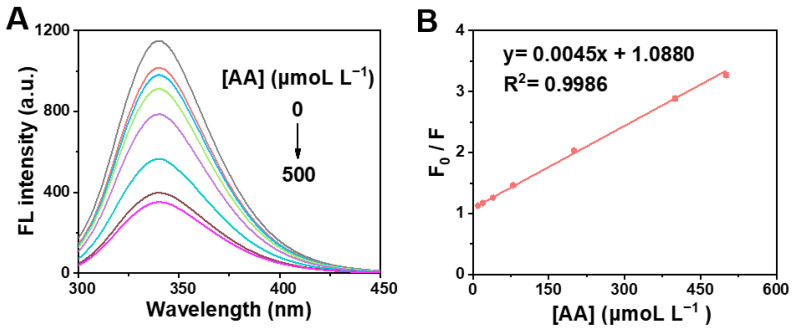
(**A**) Fluorescence emission spectra of BSA in the presence of various concentration of AA (0, 10, 20, 40, 80, 200, 400 and 500 μmoL L^−1^) at 298 K. (**B**) Corresponding working curve for AA detection.

Given the AA-caused quenching effect on the clusteroluminescence of BSA, we developed a simple and fast fluorescent detection for AA. To verify the feasibility, the fluorescent AA assay method was performed for the detection of different concentrations of AA. As illustrated in Figure 8A, with the increase in AA concentration, the corresponding fluorescence intensity of the BSA solution gradually weakened at room temperature (298 K). When the concentration of AA gradually increased from 10 to 500 μmoL L^−1^, the fluorescence intensity gradually decreased. F_0_/F shows a good linear relationship with various concentrations of AA within the range from 10 to 500 μmoL L^−1^ (Figure 8B), and the linear regression equation was y = 0.0045x + 1.0880 (R^2^ = 0.9986). The limit of detection (LOD) was estimated by the typical formula:

LOD = S/N × σ/S(7)

where S/N is the signal-to-noise ratio and is generally set to 3; σ is the standard deviation of blank; and S is the slope of the working curve [65]. Accordingly, the LOD is 6 μmoL L^−1^ (S/N = 3). Compared with other rapid AA detection methods (Table 1), the fluorescence method developed in this work has a wider linear detection range and a lower detection limit [20,66,67,68,69]. In particular, rapid detection was achieved in a short time.

Owing to the fluorescent quenching of BSA by AA, a fluorescence sensor was constructed to detect AA. BSA as a natural fluorescent probe effectively improved the speed of the fluorescent response, which demonstrated the rapid and low-cost detection of AA. The proposed method based on the AA-induced quenching effect not only has an excellent selectivity, but also displays a wide linear range toward AA. The proposed quenching-based fluorescence biosensor holds great potential to be applied for rapid AA determination.

### 2.5. AA Analysis in Real Samples

To further evaluate the practical feasibility, we used this method to determine the content of AA in vegetables. As summarized in Table 2, this method attained the fast detection of AA. The usual spiked-recovery experiment was performed to determine the accuracy of the analysis results [70]. The average recovery was in the range of 91.9–95.0% and the relative standard deviation (RSD, *n* = 5) was within 5.03%, showing the acceptable reproducibility, repeatability of AA detection in the vegetable samples.

## 3. Materials and Methods

### 3.1. Reagents and Materials

BSA (fraction V, heat shock isolation) was purchased from Shanghai Sangon Biotech Co., Ltd. (Shanghai, China). AA was obtained from Tianjin Kermel Chem reagent Co., Ltd. (Tianjin, China). Phosphate buffer saline (PBS) was prepared in this experiment. Phe was obtained from Shanghai Yuanye Bio-Technology Co., Ltd. (Shanghai, China). Pro was purchased from the Sinopharm Chemical Reagent Co., Ltd. (Shanghai, China). Glu, Ser, Asp, Val, Leu, Thr, glucose, sucrose, fructose and GSH were purchased from Shanghai Macklin Biochemical Co., Ltd. (Shanghai, China). All amino acids are L-type. All other chemicals were of analytical grade and used without further purification. The ultrapure water (18.2 MΩ cm) was obtained by the Milli-Q purification system (Milipore, Germany).

### 3.2. Apparatus

The fluorescence assay was performed using a fluorescence spectrophotometer F-2700 (Hitachi, Japan). The corresponding wavelengths were acquired with the excitation and emission slit width of 5 nm for fluorescence detection. The scan speed was set at 300 nm min^−1^, and the delay time was set to 0 s. The pH of the buffer solution was recorded using PB-10 pH Meter (Beijing Sartorius Scientific Instruments Co., Ltd., Beijing, China) and Electronic Balance Analytique AR124CN (Changzhou Ohaus Instrument Co., Ltd., Changzhou, China). The optimization of the temperature assay was recorded using the Thermostatic Water Bath (Changzhou Yineng Experimental Instrument Co., Ltd., Changzhou, China). The ultraviolet-visible (UV-vis) absorbance signal was obtained by spectrophotometer UV-2600 (Shimazu, Kyoto, Japan), and the silt width of the ultraviolet spectrum was set to 0.2 nm. Surface tensions were measured by a drop shape analyzer DSA25S (Kruss, Germany).

### 3.3. Fluorescent Spectra and Ultraviolet-Visible Absorption Spectra Measurements

The appropriate amount of AA was added to the BSA solution, and the fluorometric determinations were performed on the F-2700 spectrophotometer at the excitation wavelength of 340 nm.

In the presence and absence of AA, the ultraviolet–visible absorption spectra of BSA was measured on the UV-2600 spectrophotometer over a wavelength range of 250–350 nm at room temperature.

### 3.4. Surface Tension Measurement

The surface tension of the solution was measured by the pendant drop method. The solution was drawn into the syringe, and the needle tube was fixed on the device for testing. Finally, the surface tension of the solution at the air–water interface and the corresponding photo were obtained by dripping.

### 3.5. Fluorescent Detection of AA

For typical quantitative analysis of AA, the different concentrations of AA were added to BSA (0.5 g L^−1^). Then, PBS (pH = 7.2) was used to dilute and fix the volume. The mixture was reacted at three different temperatures for 5 min. Finally, the fluorescence spectrum was scanned and the fluorescence intensity was measured.

The effect of pH and reaction time on the fluorescence quenching of BSA by AA was investigated by the above method, except for changing the pH (pH 6.0–8.5) or reaction time (5–70 min).

To evaluate the specificity of the proposed fluorescence sensor, some common interferents (100 μmol/L), including Glu, Phe, Ser, Asp, Val, Leu, Pro, Thr, glucose, sucrose, fructose and GSH, were used to replace AA in the above fluorescent assay method. The fluorescence signal of BSA after reaction was monitored by fluorescence spectroscopy.

### 3.6. AA Detection in Real Samples

In order to evaluate the reliability of the proposed fluorescent method based on the AA-caused fluorescent quench effect of BSA, vegetables (Chinese cabbage, Turnip and Zizania latifolia) purchased from a local supermarket (RT-Mart) were employed as the actual samples. Prior to AA detection, different vegetables (100 g) were pretreated by crushing to a homogeneous state, diluting with deionized water and centrifuging at 3000 rpm. After the supernatant was filtered twice by filter membranes (0.45 and 0.22 μm), the filtrate (5 mL) was added into the BSA solution and the mixture was shaken well for testing. Subsequently, the sample solutions were measured by the fluorescence spectrometer (λ_ex_ = 288 nm, λ_em_ = 340 nm) for AA analysis. The experiment of each sample was independently tested for three times.

## 4. Conclusions

In summary, BSA was used as a natural protein clusteroluminescence probe for detecting AA. The clusteroluminescence properties of BSA were researched, and AA had obvious quenching effect on the clusteroluminescence of BSA. The proposed method of AA detection is very simple and the procedure of fluorescence quenching can be completed rapidly within 5 min. Moreover, the fluorescence intensity of BSA decreases linearly with the concentration of AA in the range of 10–500 μmoL L^−1^. The LOD of AA achieved 6 μmoL L^−1^, indicating an acceptable sensitivity. Therefore, the fluorescence AA assay method based on the fluorescence quenching of BSA shows simple operation, fast response, wide linear range, acceptable sensitivity and good selectivity. Furthermore, it was speculated that the BSA-AA binary system was a static quenching process by calculating the Stern–Volmer equation, the Lineweaver–Burk equation and the Van't Hoff equation. Overall, this work exemplifies the promising aspects of the fluorescence quenching effect for AA rapid detection, and pioneers the development of CTE effect of natural biomacromolecules. Looking forwards, this proposed strategy will find widespread uses in the analytical chemistry. The proposed quenching mechanism will provide a reference for studying the interaction between clusteroluminogens and other molecules.

## Figures and Tables

**Table 1:** Comparison of AA sensor detection with other rapid methods.

Analytical Methods	Material ^1^	Real Samples	Detection Time (min)	Linear Range (μmoL L^−1^)	LOD (μmoL L^−1^)	References
Electrochemistry	Cu HC	drugs	-	5–40	1.66	[20]
Electrochemistry	PANI/rGO	-	-	25–200	20	[66]
Colorimetry	Rh NSs	-	-	20–200	6.63	[67]
Colorimetry	Pd-Pt-Ir	-	-	25–800	11.7	[68]
Fluorescence	CD-QD@SiO_2_	fruit juice	-	0–70	3.17	[69]
Fluorescence	BSA	vegetable	5	10–500	6	This work

^1^ HC, hybrid composite; PANI/rGO, polyaniline/graphene oxide; NSs, nanosheets.

**Table 2:** Determination of AA in Real Samples.

Analytes	Concentration (μmoL L^−1^)	Added (mg/100 g)	Found (μmoL L^−1^)	Recovery (%)	Average Recovery (%)	RSD (%, *n* = 5)
Chinese cabbage	16.3	5.0	20.8	90.0	95.0	4.35
		15.0	30.3	93.3		4.09
		50.0	67.1	101.6		4.73
Turnip	9.7	3.5	12.9	91.4	92.5	4.49
		9.0	18.0	92.2		4.32
		30.0	37.9	94.0		4.81
Zizania latifolia	3.8	1.5	5.2	93.3	91.9	5.03
		4.0	7.5	92.5		4.79
		12.0	14.6	90.0		4.67

## Data Availability

The data that support the findings of this study are available from the corresponding author upon reasonable request.

## Sample Availability

Samples of the compounds are not available from the authors.
